# Mechanical ventilation of patients in helicopter emergency medical service transport: an international survey

**DOI:** 10.1186/s13049-020-00801-1

**Published:** 2020-11-18

**Authors:** Peter Hilbert-Carius, Manuel F. Struck, Veronika Hofer, Jochen Hinkelbein, Leif Rognås, Jörn Adler, Michael D. Christian, Thomas Wurmb, Michael Bernhard, Björn Hossfeld

**Affiliations:** 1 BG Klinikum Bergmannstrost Halle gGmbH, Department of Anesthesiology, Intensive Care, Emergency Medicine and Pain Therapy, and HEMS “Christoph 84” and “Christoph 85”, DRF-Luftrettung, Halle (Saale), Germany; 2 Department of Anesthesiology and Intensive Care Medicine, and HEMS “Christoph 33” and “Christoph 71” Senftenberg, University Hospital Leipzig, Leipzig, Germany; 3 Department of Anesthesiology, University Hospital Regensburg, Regensburg, Germany; 4 Department of Anesthesiology and Intensive Care Medicine, and HEMS “Christoph Rheinland”, University Hospital Cologne, Cologne, Germany; 5 Danish Air Ambulance, Aarhus, Denmark; 6 Luxembourg Air Rescue A.s.b.l., Sandweiler, Luxembourg; 7 London’s Air Ambulance, Barth’s Health NHS Trust, London, UK; 8 Department of Anesthesiology, University Hospital Würzburg, Würzburg, Germany; 9 Emergency Department, University Hospital Düsseldorf, Düsseldorf, Germany; 10 Federal Armed Forces Hospital, Ulm, Department of Anesthesiology, Intensive Care Medicine, Emergency Medicine and Pain Therapy, and HEMS “Christoph 22” Ulm, Ulm, Germany

## Abstract

**Background:**

Mechanical ventilation in helicopter emergency medical service (HEMS) environments is a procedure which carries a significant risk of complications. Limited data on the quality and performance of mechanical ventilation in HEMS are available in the literature.

**Method:**

We conducted an international survey to evaluate mechanical ventilation infrastructure in HEMS and collect data of transported ventilated patients. From June 20–22, 2019, the participating HEMS bases were asked to provide data via a web-based platform. Vital parameters and ventilation settings of the patients at first patient contact and at handover were compared using non-parametric statistical tests.

**Results:**

Out of 215 invited HEMS bases, 53 responded. Respondents were from Germany, Denmark, United Kingdom, Luxembourg, Austria and Switzerland. Of the HEMS bases, all teams were physician staffed, mainly anesthesiologists (79%), the majority were board certified (92.5%) and trained in intensive care medicine (89%) and had a median (range) experience in HEMS of 9 (0–25) years. HEMS may provide a high level of expertise in mechanical ventilation whereas the majority of ventilators are able to provide pressure controlled ventilation and continuous positive airway pressure modes (77%). Data of 30 ventilated patients with a median (range) age of 54 (21–100) years and 53% male gender were analyzed. Of these, 24 were primary missions and 6 interfacility transports. At handover, oxygen saturation (*p* < 0.01) and positive end-expiratory pressure (*p* = 0.04) of the patients were significantly higher compared to first patient contact.

**Conclusion:**

In this survey, the management of ventilated HEMS-patients was not associated with ventilation related serious adverse events. Patient conditions, training of medical crew and different technical and environmental resources are likely to influence management. Further studies are necessary to assess safety and process quality of mechanical ventilation in HEMS.

**Trial registration:**

The survey was prospectively registered at Research Registry (researchregistry2925).

## Introduction

The management of ventilated patients in helicopter emergency medical service (HEMS) has the potential for severe and life-threatening complications that may worsen critical conditions [1, 2]. These complications may include deterioration of the patients’ condition due to dynamic character of the underlying diseases or injuries themselves, as well as iatrogenic complications caused by inappropriate ventilation, unsuitable level of anesthesia, inadequate muscle relaxation, and loss of intravenous lines, chest tubes, or monitoring [3]. The need for mechanical ventilation per se is an indicator of the severity of diseases and injuries, and thorough monitoring through all steps of transportation is necessary to prevent complications [1, 2]. Data on detailed process management of ventilated HEMS patients are scarce. Recent studies suggested that real-world handling of these high-risk patients may differ from desirable standards and often depends on the discretion of the attending HEMS team [4–6].

The aim of the survey was to assess the characteristics, medical crew qualifications, and ventilator equipment of the participating HEMS bases. Furthermore, ventilator management in transported HEMS patients should be investigated regarding differences between first patient contact and handover, possible complications, handover management and safety.

## Materials and methods

The study was an online survey, without intervention and without data enabling to identify an individual HEMS team therefore [7]. The requirement for ethical approval was waived following review by the Ethical commission of the Medical Faculty of Würzburg, Germany (ID: 20171024 01). The survey complied with European data protection regulations and was prospectively registered on Research Registry (researchregistry2925). Participants were invited in a pan-European online announcement including three e-mail reminders. The announcement of the survey was sent to medical directors and HEMS bases across Europe (S1). The E-mail-distribution list was obtained from national HEMS providers and supplemented by personal contacts of the authors. The Participating HEMS teams were analyzed using an online questionnaire on three consecutive days June 20–22, 2019 (S2).

Statistical analysis was carried out mainly descriptively. Data are presented as number (%), mean (SD), or median (range) as appropriate. Regarding comparisons of vital parameters, the Mann Whitney U test was used to compare continuous or ordinal data of two independent groups. This non-parametric statistical method was used for comparing these groups because it compares the ranks and not the crude numbers without the need for a normal distribution or equal variance of the data, and protect against small number of events. A *p*-value of less than 0.05 was considered significant and all computations were performed using GraphPad Prism 8 for Windows (GraphPad Software 2020, LLC).

## Results

Two hundred and fifteen HEMS bases from 13 European countries were invited to take part in the survey (S3). Of these, 53 HEMS bases completed the survey (25%), which were included in the analysis. Within the survey period, 171 missions were performed by the participating HEMS bases.

### Characteristics of participants

Participating HEMS teams were from Germany (*n* = 26), Denmark (*n* = 16), United Kingdom (*n* = 6), Luxemburg (*n* = 2), Austria (*n* = 2) and Switzerland (*n* = 1). HEMS availability was quoted 51% 24-h and 45% until sunset (4% no response). Helicopter types were quoted Airbus H135 and predecessors 55%, Airbus H145 and predecessors 26%, McDonnell Douglas (MD) 902 13%, and Agusta Westland DaVinci 2 and 4% other types. Pilot configuration was quoted 70% single pilot, 13% dual pilot, and 17% dual pilot depending on time of day. A median (range) of 4 (0–8) HEMS mission per day were performed during the study period by the participating HEMS teams.

### Medical crew qualification

Medical team configuration was physician staffed in all respondents (physician/paramedic 92.5% and physician/flight nurse 7.5%). Three respondents (10%) had an additional trainee or resident physician on board. Most physicians had a median (range) experience in HEMS of 9 (0–25) years, were anesthesiologists (79%), board certified (92%), and had a special ICU training (89%).

### Ventilator equipment

Helicopter ventilators were able to provide only volume-controlled ventilation (VCV) in 23% of the services, whereas 77% could provide pressure control ventilation (PCV) and continuous positive airway pressure (CPAP) ventilation in addition to VCV. The most common used ventilator in the VCV only group was the Oxylog 2000 (Dräger Lübeck, Germany) and in the PCV group the Oxylog 3000 series (Dräger Lübeck, Germany). Hamilton T1 (Hamilton Medical AG, Bonaduz, Switzerland) was used in less the 10% of the participating HEMS bases. Bag/mask ventilation (BMV) was quoted as ventilator backup in all cases (55% including O_2_-demand option) while six respondents (11%) reported carrying an additional ventilator on board. Two teams perform blood gas analysis on board. A roll-in stretcher was available on 34% of the aircrafts.

### Patients’ characteristics

A total of 30 ventilated patients with a median (range) age of 54 (21–100) years were transported. During the transport, the HEMS physician was accompanied in the cabin by the paramedic/flight nurse in 34% of the cases.

Sixteen patients (53%) were male. Eighty percent (*n* = 24) of the included patients were from primary missions while six patients underwent interfacility transport (five patients from intensive care unit, ICU, and one patient from emergency department, ED). Fifteen patients (50%) suffered from trauma, seven (23%) from cardiac arrest of whom four already had a return of spontaneous circulation (ROSC) following out-of-hospital cardiopulmonary resuscitation (CPR), while one underwent helicopter transport with ongoing mechanical chest compressions using an automatic chest compression device (ACCD).

Other diagnoses of patients were neurologic/neurosurgical (*n* = 6), acute respiratory distress syndrome (ARDS, *n* = 2), and burns (*n* = 1). Airway management was performed by the HEMS-team in 18 cases (60%) of which all patients underwent tracheal intubation except one patient with a supraglottic airway device (laryngeal tube). Mechanical ventilation was performed using volume-controlled ventilation in 17 (59%) and pressure-controlled ventilation in 12 (41%) patients with a median (range) tidal volume of 6 (4-7) ml/kg body weight, whereas mode of ventilation was missing in one patient. Adaptive support ventilation (ASV) or pressure support ventilation (PSV) was not used at all during transport.

Data upon vital functions and ventilator settings at first patient contact and at handover are provided in Table 1. Two patients (7%) deteriorated during flight due to underlying critical conditions as reported by the respondents. 97% of all patients had capnography monitoring (etCO_2_) during transport with missing information in one patient. Pulse-oximetry (SpO_2_) was measurable in 27 patients (90%) while in the remaining three cases, patients were in a state of circulatory shock without sufficient peripheral perfusion for SpO_2_ measurement. At the hospital helipad, a hospital ventilator was provided in nine cases (31%), oxygen was provided in 20 cases (69%), and a medical receiving team was provided in another 20 cases (69%). Ventilation during transfer from the helipad to the receiving department was performed using the helicopter ventilator in 25 patients (83%), using a hospital ventilator in three patients (10%) and using manual bag-ventilation in one patient (3%). During transfer to the receiving department, the HEMS team carried an emergency bag / backpack in 21 cases (72%), a BMV in 27 cases (93%) and ACCD in two cases (ACCD being active in one case, as described earlier). Handover was carried out in the ED in 19 patients (65%), in the ICU in six patients (21%) and in the percutaneous coronary intervention (PCI) suite in four patients (14%).

**Table 1:** Vital parameters and ventilation settings at first patient contact and at handover

	Values at first contact	Values at handover	***p***-value
**HR [1/min]**			
median (range)	97 (0–220)	96 (0–174)	0.51
mean ± SD	99 ± 53.7	95 ± 32.7	
	*N = 30*	*N = 30*	
**SBP [mmHg]**			
median (range)	159 (130–189)	120 (90–175)	0.14
mean ± SD	159 ± 41.7	124 ± 22.5	
	*N = 2*	*N = 25*	
**RR [1/min]**			
median (range)	14 (0–22)	14 (0–20)	0.5
mean ± SD	13.3 ± 5	14.5 ± 1.9	
	*N = 28*	*N = 29*	
**SpO**_**2**_ **[%]**			
median (range)	95 (0–100)	99 (0–100)	< 0.001*
mean ± SD	80.3 ± 25	94.7 ± 18.7	
	*N = 26*	*N = 28*	
**EtCO**_**2**_ **[mmHg]**			
median (range)	36 (0–60)	36 (19–67)	0.68
mean ± SD	34.5 ± 13.5	37.6 ± 9.5	
	*N = 23*	*N = 29*	
**PEEP [cmH**_**2**_**O]**			
median (range)	5 (0–8)	5 (0–10)	0.04*
mean ± SD	4.4 ± 2.6	5.9 ± 1.8	
	*N = 27*	*N = 29*	
**PiP [cmH**_**2**_**O]**			
median (range)	22 (0–40)	22 (16–45)	0.68
mean ± SD	23 ± 8	22.5 ± 5.7	
	*N = 24*	*N = 27*	
**TV [ml]**			
median (range)	500 (350–600)	500 (360–700)	0.55
mean ± SD	495 ± 65.6	495 ± 74.6	
	*N = 21*	*N = 27*	
**TV/BW [ml/kg]**			
median (range)	6 (4–7)	6 (4–7)	0.5
mean ± SD	5.7 ± 1.6	6 ± 0.98	
	*N = 21*	*N = 27*	

*results statistically significant

*HR* Heart rate, *SBP* Systolic blood pressure, *RR* Respiratory rate, *SpO*_*2*_ Oxygen saturation, *EtCO*_*2*_ Expiratory CO_2_, *PEEP* Positive end-expiratory pressure, *PiP* Positive inspiratory pressure, *TV* Tidal volume, BW –

Patients’ devices and catheters included peripheral intravenous (IV)-lines in all but two patients (94%) whereas one had an intraosseous (IO) access and another a central venous catheter (CVC) and no peripheral IV line. Six patients had a CVC and peripheral IV-lines. A single IV-line was present in five (17%) patients, two IV-lines in 22 (73%) patients, and three IV-lines in one (3%) patient. A CVC was present in seven patients (23%) (*n* = 2 in the internal jugular vein, *n* = 1 in the subclavian vein, *n* = 4 in the femoral vein). Invasive arterial blood pressure measurement was carried out in five patients (*n* = 4 of the arterial lines were in the radial artery, *n* = 1 in the femoral artery). A urinary catheter was present in six patients (20%). CVC, arterial lines and urinary catheters were present in patients undergoing interfacility transport.

Furthermore, an abdominal drain, bilateral chest tube and pelvic sling were present in one patient, each. Syringe pump devices for continuous drug administration were present in 14 patients (48%) of which one syringe pump was used in 10 patients, two and three syringe pumps in two patients, each, and four syringe pumps in one patient. Most frequent drug administration during flight was quoted analgesics (93%), neuromuscular blocking agents (45%), and vasopressors (43%).

## Discussion

Mechanical ventilation in HEMS is a frequently performed procedure although there may be different rates across different health care systems. Almost 20% of the 171 HEMS missions in our survey involved the transport of mechanically ventilated patients. In contrast to the literature, with approximately half of all mechanically ventilated HEMS patients were interfacility transports of critical care patients, the majority of ventilated patients in our survey were primary missions [8].

In general, there are only few studies on the process quality of mechanical ventilation in HEMS environments [4, 5, 9, 10]. Key results of our small study sample demonstrate that participating HEMS-teams were entirely physician-staffed and provided a high expertise in critical care management and long experience in HEMS. Included patients had no transport-related complications of airway management and ventilator use.

In the literature, critical events (i.e., inadvertent extubation, loss of IV-lines, cardiopulmonary deterioration) occur in approximately 5% of HEMS transports of patients who are critically ill, rising up to 18% when focusing on systems with paramedics as the sole health care provider conducting interfacility critical care transfers [11]. Nevertheless, fatal events during HEMS transport are reported in less than 0.2% of cases [1, 9, 12].

Regarding ventilator settings, all patients of the present survey were ventilated with low tidal volumes and almost every second patient underwent pressure controlled ventilation (PCV), and even though the evidence of low tidal volume ventilation or PCV in patients without ARDS is low the HEMS teams use ICU standards of ventilation during transport [13, 14]. This is in contrast to published literature where tidal volumes have been reported to be above 6 ml/kg body weight in 86% of patients and most patients receive volume control ventilation during transport [6]. This may be explained by the low pre-hospital availability of transport-ventilators with PCV-mode in older studies. Nowadays modern transport-ventilators with PCV-mode are widespread even in the pre-hospital setting. High FiO_2_ during HEMS transport are common and may be the result of safety measures to prevent critical desaturation and of rare ability to perform blood gas analyzes during transport [9].

End-tidal capnography monitoring has an essential value of safety during HEMS transport. First, tube dislodgement may be detected reliably, second, hypocapnia or hypercapnia can be adjusted by changing mechanical ventilation parameters, and third, it provides prognostic value in highly critical patients [15–18]. Hypocapnia secondary to hyperventilation is a frequent iatrogenic complication during pre-hospital ventilation [19]. This has been most commonly documented among patients with traumatic brain injury and occurred in up to 79% of patients [19, 20]. Pre-hospital hyperventilation and the resulting hypocapnia are associated with poor outcomes, including increased mortality rates due to cerebral vasoconstriction causing cerebral ischemia [21]. The use of pre-hospital capnography monitoring has been identified as an outcome relevant quality parameter in the German trauma registry (TraumaRegister DGU) [22]. The high percentage (> 95%) use of capnography in our survey demonstrate the perception of the mentioned problems by the HEMS-teams.

Despite the risk of ventilator–associated critical events during HEMS-transport, there may also be opposite effects in terms of improvement of ventilation and respiratory function. In our small survey, PEEP-levels were significantly higher after transportation compared with pre-transportation PEEP, but the clinical relevance of these higher PEEP-levels could not be assessed. For instance, if the change in PEEP-level caused a PEEP over the lower inflection point of the lung, this change could be clinically relevant [23].

Across multiple studies, critical care transport teams with training in complex ventilator management are associated with improved PaO_2_ after transfer [9, 10, 12]. The transporting team changed ventilator settings during transport in most patients (decreasing tidal volume, increasing PEEP, and increasing FiO2). Furthermore, the use of neuromuscular blocking drugs is a common measure to improve respiratory function under mechanical ventilation in HEMS [9, 10]. Our data confirm these findings (45% of patients) although there was a high proportion of on-scene rescue missions and rapid sequence intubation in our cohort compared with other studies.

Notably, mechanical ventilation parameters provided by HEMS teams are known to considerably influence initial hospital ventilation parameters after patient handover [6]. Besides focusing on ventilated patients, our study-sample demonstrated a high level of expertise of participating HEMS-teams across Europe. In all cases the HEMS-teams were physician-staffed with many years of HEMS-experience along with a high proportion of board certification and ICU-training. No other studies have reported this kind of data.

The results of our survey suggest that further studies are needed to confirm whether the levels of expertise and the complication rates are representative, and if different management strategies may influence patient safety and improve process quality of mechanical ventilation in HEMS.

### Limitations

In this survey, we included a small number of HEMS-teams and patients undergoing mechanical ventilation during flight. Unfortunately, the number of responding HEMS-bases was relatively low compared to the more than 200 bases invited. One possible reason for the low response rate was, that the new general European data protection regulation came in effect just a few weeks before the survey was announced and many HEMS teams were unsure regarding participation. Furthermore, the study sample represents a heterogeneous proportion of patients of on-scene emergency responses and interfacility transports. Therefore, the management goals of these patients may not be representative of the larger patient population. Participating HEMS-teams were physician-led and other team configurations (e.g. flight nurse/paramedic or paramedic/paramedic) may have had different performances [24–27]. More than half of the participating HEMS-teams were German speaking countries. Therefore, our results may not be applicable to the general HEMS-population. Regarding the transported patients, we were not able to provide detailed information on specific drugs used, or if sedatives and analgesics were administered on basis of different scoring systems (e.g. Richmond Agitation Sedation Scale or Behaviour Pain Scale). However, we provide first data on the real-world management of mechanically ventilated HEMS-patients and our results may indicate topics relevant for future studies.

## Conclusions

In this survey, the management of ventilated HEMS-patients was not associated with ventilation related serious adverse events. Patients’ conditions, training of medical crew and different technical and environmental resources are likely to influence management. Further studies involving larger sample sizes are necessary to clarify whether different management strategies may influence patient safety and improve process quality of mechanical ventilation in HEMS.

## Supplementary information

**Additional file 1.**

## Data Availability

The survey data used to support the findings of this study are available from the corresponding author upon request.

## References

[1] Singh JM, MacDonald RD, Ahghari M (2014). Critical events during land-based interfacility transport. Ann Emerg Med.

[2] Singh JM, MacDonald RD, Bronskill SE, Schull MJ (2009). Incidence and predictors of critical events during urgent air-medical transport. CMAJ..

[3] Kiss T, Bolke A, Spieth PM (2017). Interhospital transfer of critically ill patients. Minerva Anestesiol.

[4] Hilbert-Carius P, Struck MF, Hofer V, Hinkelbein J, Wurmb T, Bernhard M (2018). Transport of ventilated emergency patients from the air rescue service to the hospital destination (HOVER study) : Results of an online survey. Anaesthesist..

[5] Hilbert-Carius P, Struck MF, Hofer V, Hinkelbein J, Wurmb T, Hossfeld B (2020). Nutzung des Hubschrauber-Respirators vom Landeplatz zum Zielort im Krankenhaus. Notfall Rettungsmed.

[6] Stoltze AJ, Wong TS, Harland KK, Ahmed A, Fuller BM, Mohr NM (2015). Prehospital tidal volume influences hospital tidal volume: a cohort study. J Crit Care.

[7] Kelley K, Clark B, Brown V, Sitzia J (2003). Good practice in the conduct and reporting of survey research. Int J Qual Health Care.

[8] Eiding H, Kongsgaard UE, Braarud AC (2019). Interhospital transport of critically ill patients: experiences and challenges, a qualitative study. Scand J Trauma Resusc Emerg Med.

[9] Wilcox SR, Saia MS, Waden H, Frakes M, Wedel SK, Richards JB (2016). Mechanical ventilation in critical care transport. Air Med J.

[10] Wilcox SR, Saia MS, Waden H, Genthon A, Gates JD, Cocchi MN (2015). Improved oxygenation after transport in patients with hypoxemic respiratory failure. Air Med J.

[11] Alabdali A, Fisher JD, Trivedy C, Lilford RJ (2017). A systematic review of the prevalence and types of adverse events in Interfacility critical care transfers by paramedics. Air Med J.

[12] Uusaro A, Parviainen I, Takala J, Ruokonen E (2002). Safe long-distance interhospital ground transfer of critically ill patients with acute severe unstable respiratory and circulatory failure. Intensive Care Med.

[13] Simonis FD, Serpa Neto A, Binnekade JM, Braber A, KCM B, Writing Group for the PI (2018). Effect of a Low vs Intermediate Tidal Volume Strategy on Ventilator-Free Days in Intensive Care Unit Patients Without ARDS: A Randomized Clinical Trial. JAMA.

[14] Fichtner F, Moerer O, Weber-Carstens S, Nothacker M, Kaisers U, Laudi S (2019). Clinical guideline for treating acute respiratory insufficiency with invasive ventilation and extracorporeal membrane oxygenation: evidence-based recommendations for choosing modes and setting parameters of mechanical ventilation. Respiration..

[15] Bieler D, Horster A, Lefering R, Franke A, Waydhas C, Huber-Wagner S (2020). Evaluation of new quality indicators for the TraumaRegister DGU((R)) using the systematic QUALIFY methodology. Eur J Trauma Emerg Surg.

[16] Childress K, Arnold K, Hunter C, Ralls G, Papa L, Silvestri S (2018). Prehospital end-tidal carbon dioxide predicts mortality in trauma patients. Prehosp Emerg Care.

[17] Langhan ML, Ching K, Northrup V, Alletag M, Kadia P, Santucci K (2011). A randomized controlled trial of capnography in the correction of simulated endotracheal tube dislodgement. Acad Emerg Med.

[18] Wilharm A, Kulla M, Baacke M, Wagner F, Behnke M, Lefering R (2019). Prähospitale Kapnometrie als Qualitätsindikator der Schwerverletztenversorgung. Eine erste Auswertung aus dem TraumaRegister DGU®. Anästh Intensivmed.

[19] Davis DP, Peay J, Sise MJ, Vilke GM, Kennedy F, Eastman AB (2005). The impact of prehospital endotracheal intubation on outcome in moderate to severe traumatic brain injury. J Trauma.

[20] Helm M, Hauke J, Lampl L (2002). A prospective study of the quality of pre-hospital emergency ventilation in patients with severe head injury. Br J Anaesth.

[21] Roberts BW, Karagiannis P, Coletta M, Kilgannon JH, Chansky ME, Trzeciak S (2015). Effects of PaCO2 derangements on clinical outcomes after cerebral injury: a systematic review. Resuscitation..

[22] Horster AC, Kulla M, Bieler D, Lefering R (2020). Empirical evaluation of quality indicators for severely injured patients in the TraumaRegister DGU(R). Unfallchirurg..

[23] Amato MB, Barbas CS, Medeiros DM, Magaldi RB, Schettino GP, Lorenzi-Filho G (1998). Effect of a protective-ventilation strategy on mortality in the acute respiratory distress syndrome. N Engl J Med.

[24] Friberg ML, Rognas L (2018). Patient-tailored triage decisions by anaesthesiologist-staffed prehospital critical care teams: a retrospective descriptive study. BMJ Open.

[25] Knapp J, Haske D, Bottiger BW, Limacher A, Stalder O, Schmid A (2019). Influence of prehospital physician presence on survival after severe trauma: systematic review and meta-analysis. J Trauma Acute Care Surg.

[26] Sollid SJM, Rehn M (2017). The role of the anaesthesiologist in air ambulance medicine. Curr Opin Anaesthesiol.

[27] van Lieshout EJ, Binnekade J, Reussien E, Dongelmans D, Juffermans NP, de Haan RJ (2016). Nurses versus physician-led interhospital critical care transport: a randomized non-inferiority trial. Intensive Care Med.

