# Correction to: Mechanical ventilation of patients in helicopter emergency medical service transport: an international survey

**DOI:** 10.1186/s13049-021-00853-x

**Published:** 2021-02-17

**Authors:** Peter Hilbert-Carius, Manuel F. Struck, Veronika Hofer, Jochen Hinkelbein, Leif Rognås, Jörn Adler, Michael D. Christian, Thomas Wurmb, Michael Bernhard, Björn Hossfeld

**Affiliations:** 1 BG Klinikum Bergmannstrost Halle gGmbH, Department of Anesthesiology, Intensive Care, Emergency Medicine and Pain Therapy, and HEMS “Christoph 84” and “Christoph 85”, DRF-Luftrettung, Halle (Saale), Germany; 2 Department of Anesthesiology and Intensive Care Medicine, and HEMS “Christoph 33” and “Christoph 71” Senftenberg, University Hospital Leipzig, Leipzig, Germany; 3 Department of Anesthesiology, University Hospital Regensburg, Regensburg, Germany; 4 Department of Anesthesiology and Intensive Care Medicine, and HEMS “Christoph Rheinland”, University Hospital Cologne, Cologne, Germany; 5 Danish Air Ambulance, Aarhus, Denmark; 6 Luxembourg Air Rescue A.s.b.l., Sandweiler, Luxembourg; 7 London’s Air Ambulance, Barth’s Health NHS Trust, London, UK; 8 Department of Anesthesiology, University Hospital Würzburg, Würzburg, Germany; 9 Emergency Department, University Hospital Düsseldorf, Düsseldorf, Germany; 10 Federal Armed Forces Hospital, Ulm, Department of Anesthesiology, Intensive Care Medicine, Emergency Medicine and Pain Therapy, and HEMS “Christoph 22” Ulm, Ulm, Germany

**Correction to: Scand J Trauma Resusc Emerg Med 28, 112 (2020)**

**https://doi.org/10.1186/s13049-020-00801-1**

Following the publication of the original article [1], the authors requested to change the corresponding author from Peter Hilbert-Carius to Manuel F. Struck.

The new corresponding author Manuel F. Struck is shown in the authorship of this Correction as well as in that of the original article.
