# Supplementary material for: Mechanical ventilation of patients in helicopter emergency medical service transport: an international survey
Source: Scand J Trauma Resusc Emerg Med. 2020 Nov 18;28:112. doi: 10.1186/s13049-020-00801-1 (PMC7672415; doi:10.1186/s13049-020-00801-1)
Supplement: Supplementary file 1 — Additional file 1. [file 13049_2020_801_MOESM1_ESM.docx]

***S1) Announcement of the survey***

**Safety and complication rates of helicopter emergency medical service transport of ventilated patients – the HOVER II study**

**Announcement of call for participation**

**Potential participants:**

Helicopter Emergency Medical Service (HEMS) bases transporting ventilated patients independent to mission (no matter if rescue mission or inter-hospital transfer)

**Status**:

In Set Up

**Trial registry**:

Research Registry (www.researchregistry.com): researchregistry2925.

**Purpose:**

Scientific research has shown that the management of ventilated patients during HEMS mission may vary considerably. The handover management of ventilated patients is particularly prone for potential complications that may deteriorate already critical patients conditions. According to a recent survey, only approximately 50% of the participants frequently carried the helicopter ventilator and emergency equipment during patient transport to the ER. Depending on the situation, more than 90% of the respondents used the helicopter ventilator and emergency backpack during the transport. The collection of the HEMS team by a hospital team at the helipad was appreciated by the majority of participants.

The purpose of this study is to assess real-world management of ventilated HEMS patients in an observational point-prevalence survey.

For patients there will be no interventions but documentation of mission-related data. Data will be collected and analyzed anonymously via an online database. The need for written informed consent was waived by the Ethical commission of the University of Würzburg, Germany.

We wish to study all patients of participating HEMS bases who are transported under ventilator use. In order to maximize sample size of participants, we will analyze three consecutive days in June 2019 (20´th of June to 22´th of June). Safety will be monitored by an independent Data Monitoring and Safety Committee.

Results from this study will be published in an international journal so that this work will have worldwide benefit.

**Contact Information**

Chief Investigator: Dr. Peter Hilbert-Carius

Study Coordination: HOVER study team: Dr. Michael Bernhard, Dr. Veronika Hofer, Dr. Björn Hossfeld, Prof Jochen Hinkelbein, Dr. Manuel Struck, Prof Thomas Wurmb

Email: [peter.hilbert@bergmannstrost.de](mailto:peter.hilbert@bergmannstrost.de)

Phone: +49 (0) 345 1327716

The survey link is going to be available with the second announcement at 1´th of June (see below)

**Timeline**

**We would be very grateful if you could forward the information regarding HOVER II to chummy HEMS bases.**

***S2) Questionnaire of the survey***

**General characteristics: HEMS team**

1. **Helicopter availability:**

O until sunset

O 24h

1. **Helicopter Type:**

O Airbus (and predecessor manufacturers)_____,

O Bell____,

O MD____,

O Sikorsky____,

O Agusta____

O other (specify):____

1. **Configuration:**

O single pilot

O dual pilot

O depending on time of day

1. **HEMS team structure:**

O Physician/Paramedic

O Physician/Flight nurse

O Flight nurse/Paramedic

O other (specify):­­____

1. **HEMS experience medical team leader:**

O Years

1. **Physician training (main area of expertise):**

O N/A

O emergency medicine

O anaesthesia

O intensive care

O medical

O surgeon / traumatologist

O other (specify:___)

1. **Physician board certification:**

O N/A

O yes

O no

1. **Physician special ICU training:**

O N/A

O yes

O no

1. **Persons on board total:**

O number

1. **Persons in cabin: number**

O number

1. **During flight in cabin with the patient:**

O paramedic

O flight nurse

O physician

O other (specify): ­­­­__

1. **HEMS ventilator:**

O VCV / IMV / IPPV only

O PCV / BIPAP possible

O CPAP / ASB / NIV possible

O specify manufacturer and type: _____

1. **HEMS ventilator backup:**

O BMV only

O BMV with O2-demand option

O additional ventilator

1. **Ability to take Blood-Gas-Analysis during flight:**

yes / no

1. **HEMS stretcher:**

O roll in stretcher

O non roll in stretcher

O depending on mission

1. **HEMS missions today:**

O total number:

O number of ventilated patients:

**If you have transported a ventilated patient today please complete the following CRF! Use a separate CRF for every ventilated patient if you have transported more than one of these patients.**

**CaseReportForm (CRF) Patient**

- **SEX:**

O male

O female

- **AGE:**

O years

- **WEIGHT:**

O kg

- **HEIGHT:**

O cm

- **APPROACH:**

O ICU

O ED

O SCENE

- **CONTEXT:**

O Trauma

O Burns

O Neurology

O Cardiothoracic

O ARDS

O post CPR

O post-surgery

O paediatric

O other (specify):_____

- **ETI:**

O HEMS

O OTHER EMS

O HOSPITAL

- **INSTRUMENTATION:**
- AIRWAY: O Endotracheal Tube (oral or nasal) O Tracheal Cannula O Supraglottic Airway
- IntraVenousLine: (number)
- CVC: O jugular V. O subclavian V. O femoral V. O none
- ArterialLine: O rad O fem O other O none
- ChestTube: O unilateral O bilateral O none
- UrinaryCatheter: O Yes O No
- OTHER DEVICE (specify):____
- **PERFUSOR (syringe-pump):**

O number

- **DRUGS:**

O Vasopressor

O Inotrope

O Sedative

O Analgesic

O Other

- **SBP (mmHg)**

O at admission___

O at handover_____

- **HR (bpm)**

O at admission___

O at handover_____

- **SAO2 (%)**

O at admission___

O at handover_____

- **ETCO2 (mmHg)**

O at admission___

O at handover_____

- **Mode of ventilation at admission:**

O VCV (IPPV, SIMV)

O PCV (BIPAP, PSV, BiLevel, APRV)

O NIV

- **Mode of ventilation at handover:**

O VCV (IPPV, SIMV)

O PCV (BIPAP, PSV, BiLevel, APRV)

O NIV

- **Patient paralysed during HEMS transport:** yes / no
- **PIP (mmHg)**

O at admission___

O at handover_____

- **PEEP (mmHg)**

O at admission___

O at handover_____

- **RF (f/min)**

O at admission___

O at handover_____

- **TV (ml)**

O at admission___

O at handover_____

- **Target Hospital - Availability of… at landing site:**

O O2

O Ventilator

O Welcome team at landing site

- **Handover place (end of HEMS responsibility):**

O Landing site

O ED

O ICU

O OR

- **Vantilator used during transfer from landing site to hospital:**

O HEMS ventilator

O hospital ventilator

O BMV only

- **Prophylactic safety measures during transfer from landing site to hospital:**

O BMV

O emergency backpack

- **Adverse events during transfer- loss of:**

O tube

O line

O device failure

O deterioration of patient condition

O none

**Thank you for your participation!**

***S3) The announcement of the survey was send to HEMS bases of the following countries:***

- **Austria**
- **Denmark**
- France
- Finland
- **Germany**
- Italy
- **Luxemburg**
- Netherland
- Norway
- Poland
- **Switzerland**
- Sweden
- **United Kingdom**

HEMS bases of countries printed in bold type participated in the survey.
